# Food Insecurity among Homeless Adults with Mental Illness

**DOI:** 10.1371/journal.pone.0159334

**Published:** 2016-07-20

**Authors:** Milad Parpouchi, Akm Moniruzzaman, Angela Russolillo, Julian M. Somers

**Affiliations:** Somers Research Group, Faculty of Health Sciences, Simon Fraser University, Burnaby, British Columbia, Canada; British Columbia Centre for Excellence in HIV/AIDS, CANADA

## Abstract

**Background:**

The prevalence of food insecurity and food insufficiency is high among homeless people. We investigated the prevalence and correlates of food insecurity among a cohort of homeless adults with mental illness in Vancouver, British Columbia, Canada.

**Methods:**

Data collected from baseline questionnaires in the Vancouver At Home study were analysed to calculate the prevalence of food insecurity within the sample (n = 421). A modified version of the U.S. Department of Agriculture’s Adult Food Security Survey Module was used to ascertain food insecurity. Univariable and multivariable logistic regression were used to examine potential correlates of food insecurity.

**Results:**

The prevalence of food insecurity was 64%. In the multivariable model, food insecurity was significantly associated with age (adjusted odds ratio [aOR] = 0.97; 95% CI: 0.95–0.99), less than high school completion (aOR = 0.57; 95% CI: 0.35–0.93), needing health care but not receiving it (aOR = 1.65; 95% CI: 1.00–2.72), subjective mental health (aOR = 0.97; 95% CI: 0.96–0.99), having spent over $500 for drugs and alcohol in the past month (aOR = 2.25; 95% CI: 1.16–4.36), HIV/AIDS (aOR = 4.20; 95% CI: 1.36–12.96), heart disease (aOR = 0.39; 95% CI: 0.16–0.97) and having gone to a drop-in centre, community meal centre or program/food bank (aOR = 1.65; 95% CI: 1.01–2.68).

**Conclusions:**

The prevalence of food insecurity was extremely high in a cohort with longstanding homelessness and serious mental illness. Younger age, needing health care but not receiving it, poorer subjective mental health, having spent over $500 for drugs and alcohol in the past month, HIV/AIDS and having gone to a drop-in centre, community meal centre or program/food bank each increased odds of food insecurity, while less than high school completion and heart disease each decreased odds of food insecurity. Interventions to reduce food insecurity in this population are urgently needed.

## Introduction

Food insecurity, which has been defined as “limited or uncertain availability of nutritionally adequate and safe foods or limited or uncertain ability to acquire acceptable foods in socially acceptable ways” [1], poses a major global public health concern. In Canada, the prevalence of experiencing food insecurity among households was estimated to be 8.3% in 2011 to 2012, equating to about 1.1 million households [2]. Food insecurity has been found to be significantly associated with poor dietary quality [3], hypertension [4], diabetes [5] and multiple chronic health conditions [6].

Homeless people are disproportionately affected by food insecurity and food insufficiency. In contrast to the definition of food insecurity presented above, food insufficiency more narrowly focuses on the lack of food and has been defined as “an inadequate amount of food intake due to a lack of money or resources” [7]. For example, a secondary analysis conducted in Canada found that 46% of those reporting current homelessness also reported being food insufficient [8]. Baggett et al. [9] reported a food insufficiency prevalence of 25.4% among a sample of homeless adults in the U.S., which was six times that of the national average found in a previous study [10]. Among those chronically homeless, the prevalence was 32.3% [9]. In another nationally representative sample of homeless and unstably housed adults in the U.S., 61.1% of respondents reported having an inadequate amount of food or were unable to get the type of food they preferred. Approximately 39% of the participants reported an inability to afford food when hungry in the previous month [11]. The prevalence of food insecurity has also been reported at considerably high rates (over 53%) among homeless and precariously housed adults living with HIV [12,13]. Unsurprisingly, homelessness has been found to be significantly associated with food insecurity or food insufficiency in the relevant literature, using adjusted models [8,14,15].

Several studies have reported variables independently associated with food insecurity or insufficiency among adult homeless populations. Lee and Greif [11] found that being a woman, younger age, lower income, street homelessness and the number of times homeless were each significantly associated with higher food insecurity. Food insecurity or food insufficiency has also been found to be significantly associated with health care utilisation, including emergency department use, hospitalisation and having an unmet need for medical or mental health care [9,13,16]. In terms of health status and physical health conditions, significant associations have been found between food insecurity and poor self-reported physical health and acute and chronic physical health problems [11,12]. Hamelin and Hemel reported significant associations between food insufficiency and poor or fair self-rated health, heart disease and having multiple chronic conditions among a nationally representative sample of adults attending various services for homeless individuals [8]. Problems with substance use or mental health and poor self-reported mental health have been found to be independently associated with food insecurity [11,12]. Food insufficiency has also been found to be associated with having poor mental health among individuals using services for the homeless [8]. Within homeless populations, those with mental illness may have greater challenges meeting subsistence needs than those without mental illness [17].

Homelessness may serve as a contextual barrier to food security in a variety of ways. Competing demands such as securing shelter, income or other subsistence needs can compromise the ability to secure an adequate amount and quality of food [18,19]. More proximal pathways include not having storage space and cooking appliances/facilities to prepare meals [18,20–22]. Additionally, homeless individuals and families located in inner city neighbourhoods face physical or financial barriers to accessing foods of sufficient quantity and nutritional quality. Structural barriers include the distance required to travel to a grocery store [23] and low-income [18,19,21,23], which can restrict the ability to purchase healthier foods [21]. Substance use is also prevalent among homeless and unstably housed individuals [24], which may further restrict available funds, and hence the ability to purchase foods [12]. To secure food, some homeless individuals may resort to extreme measures, such as sex exchange and theft [22,25].

Few peer-reviewed studies have quantitatively examined correlates and potential consequences of food insecurity among homeless adults, using adjusted models. Existing relevant research has predominantly focused on homeless adults living with HIV [12,13,26,27] or on a specific component of food insecurity (e.g. food insufficiency) [9]. An even greater paucity of research exists among homeless adults with mental illness, despite reports of significant associations between food insecurity and poorer mental health among homeless groups [11,12]. Research examining the prevalence and risk factors of food insecurity among homeless adults with mental illness is important for determining the scope of the problem and for interventions to be able to identify those most at risk. To our knowledge, the present study is the first to investigate correlates of food insecurity in a cohort of homeless adults with mental illness. We hypothesised that the prevalence of food insecurity would be substantially greater in our sample than the Canadian general population. Based on previous research, we further hypothesised that the following factors would be independently associated with food insecurity: being a woman, younger age, lower income, duration of homelessness, sex work, theft, hospital admission, emergency room (ER) visit, needing health care but not receiving it, meal program attendance, severity and quantity of mental disorder, severity and frequency of substance use, amount of money spent on substances, poorer self-reported physical, mental and overall health, heart disease, diabetes and HIV/AIDS. Due to a lack of sufficient research needed to sustain the development of a causal model and greater conceptual understanding of the directional relationship between food insecurity and independent variables, our results are intended to be exploratory.

## Methods

### Participants and sampling

Data were collected as part of the Vancouver At Home (VAH) study, involving two randomised controlled trials of Housing First interventions among homeless mentally ill adults (n = 497) in Vancouver, British Columbia (Current Controlled Trials: ISRCTN57595077; http://www.isrctn.com/ISRCTN57595077 and ISRCTN66721740; http://www.isrctn.com/ISRCTN66721740). VAH is one site of a larger multi-site study of Housing First interventions across Canada [28]. Details of the Vancouver trials have been published [29]. Briefly, participant eligibility included: being at least 19 years of age, being absolutely homeless or precariously housed and having a mental disorder. Potential participants were referred to the study between October 2009 and April 2011 by a variety of agencies (n = 40), such as shelters and mental health teams, serving homeless mentally ill individuals. Eligibility of potential participants was first discussed with the referral agency representative, and, if seemingly eligible, the participant was invited to an in-person interview, conducted by trained staff, for confirmation of eligibility and obtainment of written informed consent. Once deemed eligible, willing participants were enrolled in the study followed by a baseline interview. Participants received a cash honorarium of $35 for the baseline interview. Ethics approval for VAH was sought and approved by the institutional ethics review boards of Simon Fraser University and the University of British Columbia. For the present analyses, only data from the baseline questionnaires were included.

### Variables of interest

All questionnaires were interviewer-administered, and many were also pretested for comprehension [30]. More details about the variables and measures listed below, as well as other measures used in VAH have been published elsewhere [28,29]. Food insecurity in the past month was the primary outcome of interest and was measured using a modified version of the U.S. Department of Agriculture’s Adult Food Security Survey Module (FSSM; 2008 version) [31]. The questionnaire was modified to ensure participant comprehension, and modifications included, but were not limited to, those made by Holland, Kennedy and Hwang [32]. Examples of items include “I worried whether my food would run out before I could get more” and “Were you ever hungry but didn’t eat because you couldn’t get enough food?” The full, modified questionnaire is shown in S1 Appendix. Food insecurity was dichotomised (food secure, food insecure), and responses were scored as per the FSSM guidelines [33]. That is, a total of 0–2 and 3–10 affirmative responses to the questionnaire items were categorised as food secure and food insecure, respectively. Participants responding with “don’t know” to one or more items on the FSSM and/or those with incomplete questionnaires for any reason (e.g., declining to answer one or more items) were excluded from analyses (n = 76).

Descriptive variables and covariates for the present analyses were self-reported and selected based on relevance, as per prior research [8,9,11–13,16,21]. Socio-demographic variables included: gender (man, woman), age at randomisation (continuous years), ethnicity (Aboriginal, White, Other), education (less than high school, high school or higher), past-month income (<$800, ≥$800), age first homeless (continuous years), lifetime duration of homelessness (continuous months) and having gone to a drop-in centre/community meal centre or program/food bank in the past six months. Health care utilisation characteristics in the past six months were dichotomised (yes, no) and included: any hospital admissions, any ER visits and needing health care but not receiving it. The Mini International Neuropsychiatric Interview [34] was used to ascertain current mental disorders. The “less severe cluster of mental disorders” included having one or more of the following: panic disorder, post-traumatic stress disorder or major depressive episode. Current substance and alcohol dependence was also measured using the Mini International Neuropsychiatric Interview and was dichotomised (yes, no). The Maudsley Addiction Profile [35] was used to collect data on the following binary variables: past-month daily substance use (including alcohol), past-month use of two or more substances (including alcohol), sex work in the past month and any past-month theft. The Global Appraisal of Individual Needs, Substance Problem Scale was used to ask about money spent for drugs and alcohol in the past month [36], and this variable was dichotomised (>$500, ≤$500) based on the 80^th^ percentile of responses. Overall self-reported physical and mental health functioning were each determined by calculating their respective scores, using the Short-Form-12 Health Survey (SF-12) [37]. Overall self-rated health was also asked via the SF-12 and was then dichotomised (poor, excellent/very good/good/fair). Additionally, participants were asked if they had specific chronic physical conditions from a list of 29 to choose from. It is important to note that the present research design does not address the temporal relationship between variables. In some cases, it may be likely that variables reflect consequences of food insecurity (e.g., any hospital admissions, any ER visits, sex work and etc.). In other cases, the temporal relationship is more ambiguous (e.g., having gone to a drop-in centre/community meal centre or program/food bank, substance use variables, HIV/AIDS and etc.).

### Statistical analysis

Mann-Whitney U tests were used to test for descriptive differences between groups for continuous variables. Pearson’s chi-square or Fisher’s exact test were utilised to test for differences between groups for categorical variables, where appropriate. Univariable and multivariable logistic regression were used to estimate the relationship between covariates and food insecurity. We used “gvselect” to determine the set of independent variables with the lowest Akaike Information Criterion value for inclusion in the multivariable model [38–40]. Unadjusted and adjusted odds ratios (uOR and aOR) and 95% confidence intervals (CI) were computed with all p-values two-sided. The null hypothesis was rejected at the 0.05 level. We assessed multicollinearity for all independent variables using Variance Inflation Factor (VIF) and tolerance values [41–43]. The tolerance values for the models varied from 0.38 to 0.88, and the VIF values ranged from 1.13 to 2.65. A tolerance of ≤ 0.1 and a VIF of ≥ 10 may indicate collinearity [41,42], but all tolerance and VIF values for variables in the present analysis did not meet these collinearity cut-offs.

We also conducted a sensitivity analysis to test for differences between the participants with complete responses to the FSSM (n = 421) and those excluded (n = 76). An additional sensitivity analysis was conducted, including both participants with complete responses to all items (n = 421) and those with “don’t know” responses (n = 65) coded as negative, which equated to a total of 486 participants. The adjusted model was then re-run by using the same set of covariates included in the original multivariable model. SPSS version 22.0 (IBM Corp., Armonk, NY, USA) and STATA version 13 (StataCorp LP, College Station, TX, USA) were used to conduct statistical analyses.

## Results

A total of 497 participants completed the baseline interview. Of these participants, 421 (85%) had complete responses to all items of the FSSM. Participant characteristics are presented in Table 1. The prevalence of food insecurity was 64%.

**Table 1:** Sample characteristics by food security status.

Variable	Food secure	Food insecure	Total	P value^a^
	(n = 150; 36%)	(n = 271; 64%)	(n = 421)	
	n (%)	n (%)	n (%)	
**Socio-demographics**				
Man	108 (73)	191 (71)	299 (72)	0.792
Age at randomization (years)				
Mean (SD)	43.5 (11.7)	39.4 (10.4)	40.9 (11.0)	
Median (IQR)	44.0 (34.0–51.0)	39.0 (32.0–47.0)	41.0 (32.0–48.0)	**0.001**
Ethnicity				
Aboriginal	22 (14)	48 (18)	70 (16)	0.722
White	85 (57)	149 (55)	234 (56)	
Other	43 (29)	74 (27)	117 (28)	
Less than high school	88 (59)	149 (55)	237 (56)	0.491
Income (<$800; past month)	73 (49)	121 (45)	194 (47)	0.471
Age first homeless (years)				
Mean (SD)	33.0 (13.8)	28.7 (12.8)	30.3 (13.3)	
Median (IQR)	32.0 (20.0–42.0)	26.0 (18.0–39.0)	28.0 (19.0–41.0)	**0.004**
Lifetime duration of homelessness (months)				
Mean (SD)	56.6 (78.6)	61.0 (65.4)	59.4 (70.4)	
Median (IQR)	36.0 (12.0–72.0)	36.0 (12.0–84.0)	36.0 (12.0–84.0)	0.182
**Health care utilisation**				
Hospital admissions (past 6 months)	72 (48)	110 (41)	182 (43)	0.142
Visited Emergency Room (past 6 months)	78 (53)	160 (60)	238 (58)	0.201
Needed health care but did not receive it (past 6 months)	48 (33)	130 (48)	178 (43)	**0.002**
**Mental health**				
SF-12 mental health score				
Mean (SD)	39.0 (14.3)	32.7 (13.2)	35.0 (13.9)	
Median (IQR)	39.3 (29.1–49.6)	31.8 (23.0–42.2)	34.5 (25.0–45.7)	**<0.001**
Less severe cluster of mental disorders	66 (44)	164 (61)	230 (55)	**0.001**
2 or more mental disorders	61 (41)	148 (55)	209 (50)	**0.006**
**Substance use**				
Substance dependence	70 (47)	172 (64)	242 (58)	**0.001**
Alcohol dependence	26 (17)	74 (27)	100 (24)	**0.021**
Daily use of any substance (including alcohol; past month)	30 (20)	90 (33)	120 (29)	**0.005**
Use of multiple substances (2 or more; including alcohol; past month)	60 (40)	160 (59)	220 (53)	**<0.001**
Money spent for drugs and alcohol (past month; CAD)				
>$500	18 (12)	70 (26)	88 (21)	**0.001**
**Physical health**				
SF-12 physical health score				
Mean (SD)	45.7 (12.8)	46.2 (12.5)	46.0 (12.6)	
Median (IQR)	48.9 (36.7–56.1)	47.0 (37.0–56.8)	47.7 (36.9–56.2)	0.836
HIV/AIDS	6 (4)	29 (11)	35 (8)	**0.017**
Heart disease	14 (9)	14 (5)	28 (7)	0.096
Diabetes	11 (7)	13 (5)	24 (6)	0.275
Poor self-rated overall health	22 (15)	40 (15)	62 (15)	0.999
**Other behaviours**				
Went to any drop-in center/community meal center or program/food bank (past 6 months)	88 (60)	201 (74)	289 (69)	**0.002**
Sex work (past month)	7 (5)	31 (12)	38 (9)	**0.014**
Any theft (past month)	18 (12)	49 (18)	67 (16)	0.104

^a^Bold indicates a significant difference between food secure and insecure at p≤0.05.

The majority of the sample were men (72%), White (56%), had not completed high school (56%) and had gone to a drop-in centre, community meal centre or program/food bank in the past six months (69%). Participants had a median age of 41.0 years (interquartile range [IQR] = 32.0–48.0) at randomisation and first became homeless at a median age of 28.0 years (IQR = 19.0–41.0). The median cumulative number of months homeless was 36.0 months (IQR = 12.0–84.0). Forty-seven percent of participants reported a past-month income of less than $800, and a minority reported sex work in the past month (9%) and theft in the past month (16%).

In the past six months, 43% of participants had been admitted to hospital, a majority had visited an ER (58%) and under half (43%) reported needing health care but not receiving it.

With regard to mental illness, over half of the sample had at least one mental disorder from the “less severe cluster” (55%), and fifty percent of the participants had two or more mental disorders. The median SF-12 mental health score of participants was 34.5 (IQR = 25.0–45.7). Current substance dependence was prevalent (58%), but alcohol dependence was less common (24%). Past-month daily use of any substances, including alcohol, was reported by 29% of the sample. Use of multiple substances, including alcohol, was reported by the majority of participants (53%), and 21% reported spending over $500 on drugs and alcohol in the past month.

Specific physical health conditions, such as HIV/AIDS, heart disease and diabetes were reported by eight, seven and six percent of the sample, respectively. Poor self-rated overall health was uncommon among the sample (15%), and the median SF-12 physical health score was 47.7 (IQR = 36.9–56.2).

The unadjusted and adjusted analyses are shown in Table 2. Several factors were significantly associated with food insecurity in the unadjusted analysis and included the following: age (uOR = 0.97; 95% CI: 0.95–0.99), age first homeless (uOR = 0.98; 95% CI: 0.96–0.99), needing health care but not receiving it in the past 6 months (uOR = 1.94; 95% CI: 1.28–2.96), SF-12 mental health score (uOR = 0.97; 95% CI: 0.95–0.98), having at least one mental disorder from the “less severe cluster” (uOR = 1.95; 95% CI: 1.30–2.92), having two or more mental disorders (uOR = 1.76; 95% CI: 1.17–2.63), substance dependence (uOR = 1.99; 95% CI: 1.32–2.98), alcohol dependence (uOR = 1.79; 95% CI: 1.09–2.95), past-month daily use of any substances, including alcohol (uOR = 1.97; 95% CI: 1.22–3.16), past-month use of two or more substances, including alcohol (uOR = 2.13; 95% CI: 1.42–3.21), spending over $500 for drugs and alcohol in the past month (uOR = 2.59; 95% CI: 1.48–4.56), HIV/AIDS (uOR = 2.88; 95% CI: 1.17–7.10), having gone to a drop-in centre, community meal centre or program/food bank in the past six months (uOR = 1.99; 95% CI: 1.30–3.05) and sex work in the past month (uOR = 2.65; 95% CI: 1.14–6.17). Eight independent variables, which made up the lowest Akaike Information Criterion value, were entered into the multivariable model. Each of the eight variables was significantly associated with food insecurity: age (aOR = 0.97; 95% CI: 0.95–0.99), less than high school completion (aOR = 0.57; 95% CI: 0.35–0.93), needing health care but not receiving it in the past 6 months (aOR = 1.65; 95% CI: 1.00–2.72), SF-12 mental health score (aOR = 0.97; 95% CI: 0.96–0.99), having spent over $500 for drugs and alcohol in the past month (aOR = 2.25; 95% CI: 1.16–4.36), HIV/AIDS (aOR = 4.20; 95% CI: 1.36–12.96), heart disease (aOR = 0.39; 95% CI: 0.16–0.97) and having gone to a drop-in centre, community meal centre or program/food bank in the past six months (aOR = 1.65; 95% CI: 1.01–2.68). Results from the multivariable sensitivity analysis were similar. Also, as shown in S1 Table, there were no significant differences between participants with complete responses and those excluded for any of the characteristics chosen for inclusion in the present analyses. Participant characteristics, stratified by food security status, for all participants, including those with “don’t know responses” (n = 486) are shown in S2 Table.

**Table 2:** Univariable and multivariable logistic regression analyses of correlates of food insecurity (n = 421).

Variable	uOR^a^ (95% CI^b^)^c^	aOR^d^ (95% CI)^c^
**Socio-demographics**		
Man	0.94 (0.60, 1.47)	
Age at randomization (years)	**0.97 (0.95, 0.99)**	**0.97 (0.95, 0.99)**
Ethnicity		
Aboriginal	1.27 (0.68, 2.38)	
White	1.02 (0.64, 1.62)	
Other	Reference	
Less than high school	0.87 (0.58, 1.30)	**0.57 (0.35, 0.93)**
Income (<$800; past month)	0.86 (0.58, 1.29)	
Age first homeless (years)	**0.98 (0.96, 0.99)**	
Lifetime duration of homelessness (months)	1.00 (1.00, 1.00)	
**Health care utilisation**		
Hospital admissions (past 6 months)	0.74 (0.50, 1.11)	
Visited Emergency Room (past 6 months)	1.30 (0.87, 1.96)	
Needed health care but did not receive it (past 6 months)	**1.94 (1.28, 2.96)**	**1.65 (1.00, 2.72)**
**Mental health**		
SF-12 mental health score	**0.97 (0.95, 0.98)**	**0.97 (0.96, 0.99)**
Less severe cluster of mental disorders	**1.95 (1.30, 2.92)**	
2 or more mental disorders	**1.76 (1.17, 2.63)**	
**Substance use**		
Substance dependence	**1.99 (1.32, 2.98)**	
Alcohol dependence	**1.79 (1.09, 2.95)**	
Daily use of any substance (including alcohol; past month)	**1.97 (1.22, 3.16)**	
Use of multiple substances (2 or more; including alcohol; past month)	**2.13 (1.42, 3.21)**	
Money spent for drugs and alcohol (>$500; past month; CAD)	**2.59 (1.48, 4.56)**	**2.25 (1.16, 4.36)**
**Physical health**		
SF-12 physical health score	1.00 (0.99, 1.02)	
HIV/AIDS	**2.88 (1.17, 7.10)**	**4.20 (1.36, 12.96)**
Heart disease	0.53 (0.24, 1.13)	**0.39 (0.16, 0.97)**
Diabetes	0.63 (0.28, 1.45)	
Poor self-rated overall health	1.00 (0.57, 1.76)	
**Other behaviours**		
Went to any drop-in center/community meal center or program/food bank (past 6 months)	**1.99 (1.30, 3.05)**	**1.65 (1.01, 2.68)**
Sex work (past month)	**2.65 (1.14, 6.17)**	
Any theft (past month)	1.62 (0.90, 2.89)	

^a^uOR = unadjusted odds ratio.

^b^CI = confidence interval.

^c^Bold indicates significance at p≤0.05.

^d^aOR = adjusted odds ratio.

## Discussion

Our study results demonstrate the urgent need for food insecurity interventions among homeless adults with mental illness in Vancouver, BC. The prevalence of food insecurity in our sample was 64%. The prevalence of household food insecurity in Canada was reported to be 8.3% in 2011–2012 [2]. Our prevalence is almost eight times higher than the Canadian general household population. It was also higher than the prevalence of food insufficiency in other homeless samples [8,9] or food insecurity among homeless individuals with HIV [12,13]. This finding is a cause for public health concern given food security is an important and globally recognised human right and social determinant of health. Several variables were also independently associated with food insecurity, namely age, formal education level, needing health care but not receiving it, SF-12 mental health score, money spent on drugs and alcohol, HIV/AIDS, heart disease and having gone to a drop-in centre, community meal centre or program/food bank.

Of all socio-demographic characteristics, age and education level were each independently associated with food insecurity. Specifically, age was negatively associated with food insecurity. It may be that younger homeless adults with mental illness are more likely to be more recently homeless and lack relevant knowledge and experience to secure an adequate quality and quantity of food relative to their older counterparts. A similar observation has been made by other researchers investigating food insecurity among homeless adults [11]. However, findings from previous studies have been mixed. For example, one study of food insufficiency conducted among adults attending various services for homeless individuals found food insufficiency not to be significantly associated with age [8]. This was consistent with another study published in the same year on food insecurity among homeless and precariously housed adults living with HIV [12]. Screening programs should pay particular attention to younger homeless adults with mental illness.

On the other hand, less than high school completion was significantly associated with decreased odds of food insecurity. The potential explanation for this relationship is less apparent. Few studies have reported the specific association between education and food insecurity among those who are homeless. Weiser et al. [12] found no association between education and food insecurity among homeless and precariously housed individuals living with HIV. This finding was similar to Hamelin and Hamel’s study [8] among adults attending various services for homeless individuals. Our finding may be attributable to members of our sample with lower formal educational achievement being more likely to have cognitive disorders that would make them more eligible for social supports and services involving food insecurity that would not be provided as frequently to people with higher degrees of formal education and intellectual capability. This finding, however, needs further examination, and our results indicate that education may not be protective against food insecurity among people who are homeless with mental illness.

Needing health care but not receiving it was significantly associated with higher food insecurity in our sample. Having competing priorities has been proposed as a contributor to this association where homeless individuals’ subsistence needs, including food, can serve as barriers to access to health care [44,45]. Baggett et al. [16] further subdivided unmet need for health care into 5 different types and found that food insufficiency was significantly associated with three types: “medical or surgical care,” “mental health care” and “prescription medications.”

The mental health score of the SF-12 was negatively associated with food insecurity in the multivariable model. This is consistent with a previous study conducted among homeless individuals living with HIV in the US [12]. Moreover, as previously noted, other studies have also reported associations between poorer mental health and food insecurity or insufficiency [8,11]. Potential pathways between food insecurity and poorer mental health have been suggested [46], but further research is needed to clarify the direction of causality. Additionally, while objective assessment and diagnosis of psychopathology is important, the present findings indicate the importance of ascertaining an individual’s subjective assessment of their mental health, as this variable (and not diagnostic status per se) was related to food insecurity.

The finding that over $500 spent for drugs and alcohol was significantly associated with increased food insecurity may reflect the straightforward reality that money spent for substances cannot be used to purchase food. Interventions aiming to reduce substance use may also aid in reducing food insecurity among homeless individuals with mental illness. Interventions that employ a harm reduction approach (e.g., Housing First) may need to consider money spent on drugs and alcohol and its implications for food insecurity.

With regard to the physical health variables, HIV/AIDS status was significant; those with self-reported HIV/AIDS had over four times the odds of being food insecure compared to those without HIV/AIDS in the adjusted model. There is a high prevalence of food insecurity among people living with HIV [47] and those who are both homeless and living with HIV [12,13]. Food insecurity may be both a contributing factor to and a consequence of HIV/AIDS [48]. Our finding is concerning in light of previous research demonstrating adverse HIV-related health outcomes associated with food insecurity among homeless individuals living with HIV, including, but not limited to, low CD4 counts [12], incomplete viral suppression [26] and low antiretroviral therapy adherence [26]. Food insecurity interventions among homeless individuals with mental illness should recognise HIV/AIDS as a potential factor for increased food insecurity, and, as researchers have previously argued, there is a need for food insecurity interventions to be considered and implemented as part of broader HIV treatment and prevention programs [26].

Contrary to our expectation, heart disease was associated with decreased odds of food insecurity. This finding is also contrary to another study conducted among adults attending various services for homeless individuals, which found that food insufficiency was significantly associated with higher odds of heart disease [8]. It may be that members of our sample with knowledge of having heart disease paid greater attention to nutrition and food sufficiency, hence contributing to greater food security.

Similar to a nationally representative sample conducted in the US [11], we found attendance at a drop-in centre, community meal centre or program/food bank to be significantly associated with increased odds of food insecurity. As posited by Lee and Greif [11], meal programs may be used as an emergency resource and not necessarily as a preventive strategy. However, it may be that meal programs contribute to food insecurity. Many homeless individuals may depend on or use charitable food programs [11,21,49], but evaluations of such programs have revealed that their meals may be inadequate both in terms of the quantity/frequency [50,51] and nutritional content provided to meet their needs [50,52]. For example, the current system of food provision to individuals who are food insecure in Canada, largely provided by charitable organisations, has been characterised as inadequate and poorly coordinated [51]. More broadly, and in the case of homeless individuals with mental illness, structural interventions, such as appropriately supported housing, may have a larger impact on the reduction of food insecurity by removing or alleviating contextual barriers previously discussed (e.g., competing demands and lack of food storage space) while simultaneously providing health and social supports.

This study had several limitations. Due to the cross-sectional nature of our study design, the directionality of the relationship between food insecurity and correlates found cannot be determined. Moreover, all data were self-reported and vulnerable to bias, including recall and social desirability bias. Covariates included in our analyses may have also been underreported (e.g., HIV/AIDS, money spent on drugs and alcohol, sex work and etc.), biasing results towards the null. However, 30 participants underwent blood testing for HIV and all were negative, which was in 100% agreement with their self-reported HIV/AIDS status. The meal program attendance variable used for the present study was a compound item combining attendance at any drop-in centre with attendance at any meal program. It would be useful for future research to disaggregate this item in order to measure the association of each component with food insecurity, separately. About 15% of participants were excluded from analyses, but sensitivity analyses revealed no meaningful impacts on our results. Lastly, in the absence of a probability sample, findings may not be generalisable to other locales and require replication elsewhere.

## Conclusions

To our knowledge, this was the first study to investigate food insecurity among homeless adults with serious mental illness. The prevalence of food insecurity in our sample was extremely high, signaling the urgent need for action. Several correlates were significantly and independently associated with food insecurity: age; education; needing health care but not receiving it; subjective mental health; money spent for drugs and alcohol; HIV/AIDS; heart disease; and having visited any drop-in centre, community meal centre or program/food bank. These findings await replication, and in the meantime may be considered when screening for food insecurity among homeless mentally ill people. The nature of some of these correlates and their association with food insecurity are not intuitively obvious (e.g., education), with further research needed. Future studies utilising longitudinal designs are also needed to examine the temporal relationship between food insecurity and identified correlates among homeless adults with mental illness. Moreover, interventional research is needed to examine effective ways to reduce food insecurity in this population and improve related outcomes.

## Supporting Information

S1 Appendix Modified version of the US Department of Agriculture’s Adult Food Security Survey Module. (DOC)

S1 Table Comparison of characteristics between participants with valid responses (n = 421) and participants with missing responses (n = 76). (DOC)

S2 Table Sample characteristics by food security status (n = 486). (DOC)
