# Supplementary material for: Food Insecurity among Homeless Adults with Mental Illness
Source: PLoS One. 2016 Jul 20;11(7):e0159334. doi: 10.1371/journal.pone.0159334 (PMC4954689; doi:10.1371/journal.pone.0159334)
Supplement: S1 Appendix — (DOC) [file pone.0159334.s001.doc]

**S1 Appendix. Modified version of the US Department of Agriculture’s Adult Food Security Survey Module**

| Question | Response categories |
| --- | --- |
| 1. I worried whether my food would run out before I could get more. | Often true, sometimes true, never true, don’t know, declined |
| 2. The food that I got just didn't last, and I couldn't get more. | Often true, sometimes true, never true, don’t know, declined |
| 3. I couldn't eat balanced meals. (By balanced we mean eating different types of foods to get a wide range of nutrients e.g. fiber, vitamins, minerals). | Often true, sometimes true, never true, don’t know, declined |
| 4. Did you ever cut the size of your meals or skip meals because you couldn't get enough food? (If yes, ask 4.a.)^a^  4.a. In the last 30 days, how many days did this happen? | Yes, no, don’t know, declined  1-30 days^b^ |
| 5. Did you ever eat less than you felt you should because you couldn't get enough food? | Yes, no, don’t know, declined |
| 6. Were you ever hungry but didn't eat because you couldn't get enough food? | Yes, no, don’t know, declined |
| 7. Did you lose weight because you couldn't get enough food? | Yes, no, don’t know, declined |
| 8. Did you ever not eat for a whole day because you couldn't get enough food? (If yes, ask 8.a.)^c^  8.a. In the last 30 days, how many days did this happen? | Yes, no, don’t know, declined  1-30 days^b^ |
| 9. Would you say that the food you get is nutritious? (By nutritious we mean a variety of whole foods including fruits and vegetables) | Yes, no, don’t know, declined |
| 10. Are you usually able to get good quality food? (By quality we mean fresh and unspoiled) | Yes, no, don’t know, declined |

^a^Affirmative responses to question 4 were considered as such only if an affirmative response was also given to sub-question 4.a. Negative responses to sub-question 4.a. were added to the negative responses of question 4.

^b^Responses of ≥3 days were considered affirmative. Responses of ≤2 days were considered negative.

^c^Affirmative responses to question 8 were considered as such only if an affirmative response was also given to sub-question 8.a. Negative responses to sub-question 8.a. were added to the negative responses of question 8.
