# Supplementary material for: Food Insecurity among Homeless Adults with Mental Illness
Source: PLoS One. 2016 Jul 20;11(7):e0159334. doi: 10.1371/journal.pone.0159334 (PMC4954689; doi:10.1371/journal.pone.0159334)
Supplement: S1 Table — (DOC) [file pone.0159334.s002.doc]

**S1 Table. Comparison of characteristics between participants with valid responses (n=421) and participants with missing responses (n=76)**

| **Variable** | **Participants with valid responses**  **(n = 421)**  **n (%)** | **Participants with missing responses**  **(n=76)**  **n (%)** | **All participants**  **(n=497)**  **n (%)** | **P value^a^** |
| --- | --- | --- | --- | --- |
| **Socio-demographics** |  |  |  |  |
| Man | 299 (72) | 60 (79) | 359 (73) | 0.192 |
| Age at randomization (years)  Mean (SD)  Median (IQR) | 40.9 (11.0)  41.0 (32.0-48.0) | 40.6 (10.8)  41.0 (32.0-49.0) | 40.8 (11.0)  41.0 (32.0-48.0) | 0.942 |
| Ethnicity  Aboriginal  White  Other | 70 (16)  234 (56)  117 (28) | 7 (9)  46 (61)  23 (30) | 77 (16)  280 (56)  140 (28) | 0.259 |
| Less than high school | 237 (56) | 43 (58) | 280 (57) | 0.788 |
| Income (<$800; past month) | 194 (47) | 40 (53) | 234 (48) | 0.285 |
| Age first homeless (years)  Mean (SD)  Median (IQR) | 30.3 (13.3)  28.0 (19.0-41.0) | 30.3 (13.3)  28.0 (19.0-41.0) | 30.3 (13.3)  28.0 (19.0-41.0) | 0.999 |
| Lifetime duration of homelessness (months)  Mean (SD)  Median (IQR) | 59.4 (70.4)  36.0 (12.0-84.0) | 64.5 (70.2)  48.0 (18.0-84.0) | 60.2 (70.3)  36.0 (12.0-84.0) | 0.426 |
| **Health care utilisation** |  |  |  |  |
| Hospital admissions (past 6 months) | 182 (43) | 30 (39) | 212 (43) | 0.542 |
| Visited Emergency Room (past 6 months) | 238 (58) | 43 (58) | 281 (58) | 0.939 |
| Needed health care but did not receive it (past 6 months) | 178 (43) | 31 (45) | 209 (43) | 0.752 |
| **Mental health** |  |  |  |  |
| SF-12 mental health score  Mean (SD)  Median (IQR) | 35.0 (13.9)  34.5 (25.0-45.7) | 37.8 (12.5)  38.3 (28.8-46.5) | 35.4 (13.7)  35.6 (25.7-45.8) | 0.094 |
| Less severe cluster of mental disorders | 230 (55) | 34 (45) | 264 (53) | 0.112 |
| 2 or more mental disorders | 209 (50) | 31 (41) | 240 (48) | 0.155 |
| **Substance use** |  |  |  |  |
| Substance dependence | 242 (58) | 46 (61) | 288 (58) | 0.621 |
| Alcohol dependence | 100 (24) | 21 (28) | 121 (24) | 0.468 |
| Daily use of any substance (including alcohol; past month) | 120 (29) | 23 (30) | 143 (29) | 0.783 |
| Use of multiple substances (2 or more; including alcohol; past month) | 220 (53) | 37 (49) | 257 (52) | 0.526 |
| Money spent for drugs and alcohol (past month; CAD)  >$500 | 88 (21) | 13 (17) | 101 (21) | 0.406 |
| **Physical health** |  |  |  |  |
| SF-12 physical health score  Mean (SD)  Median (IQR) | 46.0 (12.6)  47.7 (36.9-56.2) | 45.4 (10.6)  45.0 (37.3-54.9) | 45.9 (12.3)  47.5 (36.9-55.9) | 0.446 |
| HIV/AIDS | 35 (8) | 8 (10) | 43 (9) | 0.528 |
| Heart disease | 28 (7) | 4 (5) | 32 (6) | 0.803^b^ |
| Diabetes | 24 (6) | 5 (7) | 29 (6) | 0.789^b^ |
| Poor self-rated overall health | 62 (15) | 5 (7) | 67 (13) | 0.055 |
| **Other behaviours** |  |  |  |  |
| Went to any drop-in center/community meal center or program/food bank (past 6 months) | 289 (69) | 53 (70) | 342 (69) | 0.917 |
| Sex work (past month) | 38 (9) | 5 (7) | 43 (9) | 0.483 |
| Any theft (past month) | 67 (16) | 8 (11) | 75 (15) | 0.228 |

^a^Bold indicates a significant difference between food secure and insecure at p≤0.05

^b^Fisher’s exact test used
