# Supplementary material for: Food Insecurity among Homeless Adults with Mental Illness
Source: PLoS One. 2016 Jul 20;11(7):e0159334. doi: 10.1371/journal.pone.0159334 (PMC4954689; doi:10.1371/journal.pone.0159334)
Supplement: S2 Table — (DOC) [file pone.0159334.s003.doc]

**S2 Table. Sample characteristics by food security status (n=486)**

| **Variable** | **Food secure**  **(n = 167; 34%)**  **n (%)** | **Food insecure**  **(n=319; 66%)**  **n (%)** | **Total**  **(n=486)**  **n (%)** | **P value^a^** |
| --- | --- | --- | --- | --- |
| **Socio-demographics** |  |  |  |  |
| Man | 122 (74) | 231 (73) | 353 (73) | 0.926 |
| Age at randomization  Mean (SD)  Median (IQR) | 42.8 (11.6)  44.0 (33.0-51.0) | 39.7 (10.4)  40.0 (32.0-47.0) | 40.8 (10.9)  41.0 (32.0-48.0) | **0.007** |
| Ethnicity  Aboriginal  White  Other | 22 (13)  97 (58)  48 (29) | 54 (17)  175 (55)  90 (28) | 76 (16)  272 (56)  138 (28) | 0.548 |
| Less than high school | 99 (60) | 178 (56) | 277 (57) | 0.462 |
| Income (<$800; past month) | 82 (49) | 147 (47) | 229 (48) | 0.569 |
| Age first homeless (years)  Mean (SD)  Median (IQR) | 32.9 (13.6)  32.0 (21.0-42.0) | 28.8 (12.9)  26.0 (18.0-39.0) | 30.2 (13.3)  28.0 (19.0-41.0) | **0.002** |
| Lifetime duration of homelessness (months)  Mean (SD)  Median (IQR) | 54.1 (75.9)  36.0 (12.0-72.0) | 63.2 (67.0)  42.0 (14.0-84.0) | 60.1 (70.2)  36.0 (12.0-84.0) | **0.024** |
| **Health care utilisation** |  |  |  |  |
| Hospital admissions (past 6 months) | 80 (48) | 127 (40) | 207 (43) | 0.087 |
| Visited Emergency Room (past 6 months) | 89 (55) | 186 (59) | 275 (58) | 0.331 |
| Needed health care but did not receive it (past 6 months) | 54 (33) | 149 (48) | 203 (43) | **0.002** |
| **Mental health** |  |  |  |  |
| SF-12 mental health score  Mean (SD)  Median (IQR) | 39.3 (14.2)  40.2 (29.1-49.6) | 33.3 (13.1)  32.5 (24.1-42.4) | 35.3 (13.8)  35.5 (25.7-45.8) | **<0.001** |
| Less severe cluster of mental disorders | 72 (43) | 188 (59) | 260 (54) | **0.001** |
| 2 or more mental disorders | 67 (40) | 167 (52) | 234 (48) | **0.010** |
| **Substance use** |  |  |  |  |
| Substance dependence | 83 (50) | 200 (63) | 283 (58) | **0.006** |
| Alcohol dependence | 30 (18) | 89 (28) | 119 (24) | **0.016** |
| Daily use of any substance (including alcohol; past month) | 35 (21) | 107 (34) | 142 (29) | **0.004** |
| Use of multiple substances (2 or more; including alcohol; past month) | 68 (41) | 185 (58) | 253 (52) | **<0.001** |
| Money spent for drugs and alcohol (past month; CAD)  >$500 | 19 (11) | 81 (26) | 100 (21) | **<0.001** |
| **Physical health** |  |  |  |  |
| SF-12 physical health score  Mean (SD)  Median (IQR) | 45.8 (12.4)  48.6 (36.9-56.1) | 46.0 (12.4)  47.0 (37.0-55.9) | 45.9 (12.4)  47.5 (37.0-55.9) | 0.999 |
| HIV/AIDS | 7 (4) | 35 (11) | 42 (9) | **0.012** |
| Heart disease | 15 (9) | 15 (5) | 30 (6) | 0.060 |
| Diabetes | 11 (7) | 17 (5) | 28 (6) | 0.561 |
| Poor self-rated overall health | 23 (14) | 44 (14) | 67 (14) | 0.985 |
| **Other behaviours** |  |  |  |  |
| Went to any drop-in center/community meal center or program/food bank (past 6 months) | 99 (60) | 237 (75) | 336 (70) | **0.001** |
| Sex work (past month) | 7 (4) | 35 (11) | 42 (9) | **0.011** |
| Any theft (past month) | 21 (13) | 53 (17) | 74 (15) | 0.243 |

^a^Bold indicates a significant difference between food secure and insecure at p≤0.05
